# Death from Burns

**Published:** 1890-10-11

**Authors:** 


### DEATH FROM BURNS.

It has been usual to attribute the fatal termination of extensive though superficial burns and scalds to the effects of "shock," which is, to say the least, a somewhat vague expression. Dr. Silbermann in a contribution to *Virchow's Archives* refers it to the changes morphological and vital produced in the blood corpuscles, which, together with the débris of broken up corpuscles and discs cause thrombi and blood stasis in various organs and tissues as the lungs, liver, kidneys, the walls of the alimentary canal, the brain, and the subcutaneous connective tissue, but most numerous in the lungs. The vascular obstructions and stasis in the pulmonary arteries, lead to general venous stasis with distension of the right ventricle and on the other hand marked arterial anæmia; and as a consequence internal hæmorrhage, abscesses and parenchymatous changes in the several organs.

These conditions fully explain the familiar symptoms of dyspnœa, cyanosis, coma, small pulse, spasms, lung complications and the fall of the temperature, as well as the frequent postponement of the fatal termination till after the lapse of even a week, when all danger from shock and collapse must have passed away; while the small size of the heart, the feeble resisting power of the red blood cells, and the thinness of the skin of children account for the readiness with which they succumb to comparatively slight injuries of this kind.

